# “It's a good idea, but…”: a qualitative evaluation of the GoldiCare intervention in Norwegian home care services

**DOI:** 10.3389/frhs.2024.1511772

**Published:** 2025-01-20

**Authors:** Heike Fischer, Fredrik Klæboe Lohne, Marius Steiro Fimland, Skender Elez Redzovic

**Affiliations:** ^1^ Department of Neuromedicine and Movement Science, Faculty of Medicine and Health Sciences, NTNU Norwegian University of Science and Technology, Trondheim, Norway; ^2^ The National Research Centre for the Working Environment, Copenhagen, Denmark

**Keywords:** process evaluation, implementation outcomes, qualitative analysis, goldilocks work principle, home care workers, musculoskeletal pain, occupational physical activity, workplace health promotion

## Abstract

**Background:**

Addressing high levels of physical strain among Norwegian home care workers is crucial if home care services are to continue to provide cost-effective and high-quality health care for people in their homes. Excessive physical demands may contribute to poor long-term musculoskeletal health and high sick leave rates among home care workers. Based on the Goldilocks Work Principle of redistributing an uneven distribution of physical demands to promote a working environment with a “just right” physical demands conducive to promoting long-term health, the GoldiCare intervention in home care services was conducted. The objective of this qualitative process evaluation study was to gain insights into how the implementation outcomes of acceptability, appropriateness, feasibility, adoption and fidelity, respectively, impacted the implementation of the GoldiCare intervention.

**Methods:**

We conducted ten individual interviews with operations managers and five focus group interviews with home care workers from the intervention units. Interviews were transcribed verbatim and a three step-content analysis was employed to analyze interview material.

**Results:**

Our analysis identified that although the intervention was considered broadly acceptable, there were several challenges corresponding to the dimensions of appropriateness, feasibility, adoption and fidelity. Major barriers were identified in particular with regard to appropriateness, that is underlying ways of measuring physical demands; and feasibility, that is barriers to implementing the tool. Further synthesis of these findings resulted in four core issues that need to be addressed if the GoldiCare intervention is to be successfully implemented in comparable Norwegian home care settings: proxy issues; complexity and unpredictability; organization-level issues; and operational autonomy.

**Conclusion:**

The findings provide valuable insights for future attempts to implement GoldiCare interventions in home care settings, highlighting the need to further integrate GoldiCare and other comparable types of intervention into the political, economic, sociocultural, professional, and technological context of home care services. Performed in the right way, such integration will also allow for more participatory input from those enacting such interventions.

**Trial registration:**

This clinical trial was registered on 08/05/2022 under NCT05 487027.

## 1 Introduction

Ageing populations and an associated increased demand for care services, in particular long-term care services, are well-known developments across Organisation for Economic Co-operation and Development (OECD) countries (1). Given the economic challenges surrounding institutionalized care, home care (HC) is the fastest growing type of care (2, 3). In Norway, institutional care is reserved for patients unable to live at home or patients needing urgent medical attention (4). Among Norwegians aged 80 and older, 92.4% live in private households (5). HC services offer a cost-effective way to deliver care (6, 7) and HC is often preferred by patients to institutionalized care (8–10). If political goals around “aging in place” are to be realized in the coming decades, recruiting staff and, importantly, retaining healthy staff will be crucial (6, 7, 11).

Sick leave rates among home care (HC) workers in Norway are substantially higher than the national average; at around 11% almost double the national average (12). While reasons behind sick leave rates among HC workers are multifactorial, research from Norway shows that workplace-related factors are important contributors and that physical demands may play a significant role (13). No study in Norway has focused on interventions to reduce physical strain among HC workers. A systematic review by Gebhard and Herz (14) shows that out of six international interventions that included musculoskeletal pain in their intervention outcomes, only two found significant effects. Two interventions (15, 16) restructured the way schedulers, referred to as operations coordinators in the present study, allocated the most demanding clients. However, neither study employed a randomized controlled trial (RCT) design; both used a pre-to-post design. Additionally, both interventions included multiple components, making it difficult to isolate the specific impact of the reorganization alone. Thus, despite a need to address physical demands among HC workers, little knowledge exists about how to effectively achieve this.

Physical demands among HC workers are unevenly distributed in the study context (17). Based on the Goldilocks Work Principle proposed by Holtermann et al. (18), which advocates for redistributing uneven physical demands to create a work environment with “just right” levels of physical activity that support long-term health, the GoldiCare intervention for home care services was developed and tested through a cluster-randomized controlled trial^1^ (from here on referred to as the GoldiCare intervention). The underlying rationale for the GoldiCare intervention was to redistribute weekly work demands amongst HC workers, thereby aiming to improve musculoskeletal health in HC workers. The intervention was implemented at the organizational level, in that operations managers (OMs) were to employ the GoldiCare tool. The GoldiCare tool was designed to rebalance HC workers' potentially unevenly distributed weekly workload by rotating both heavy, moderately heavy and lighter work schedules [for further details, see (19)].

Quantitative analysis of implementation outcomes shows that the GoldiCare intervention was only employed to a moderate degree (measured in terms of how many weeks the GoldiCare tool was employed) and that there was no significant improvement in terms of more balanced weekly workloads (20). This study complements these quantitative process evaluation findings by providing a qualitative in-depth understanding of the processes underlying the intervention's implementation outcomes. More specifically, the objectives of this study were: (1) to identify perceived barriers and facilitators for implementing the GoldiCare intervention; (2) to provide a qualitative supplement for better understanding the underlying processes behind the reach of the intervention; (3) to evaluate how well the intervention was implemented according to the protocol; and (4) to identify future challenges and solutions for GoldiCare interventions in the Norwegian HC sector.

## 2 Study context and theoretical framework

### 2.1 Study context

Norwegian HC services are organized and financed at municipality level, providing short-term and long-term care. Patients receiving HC services can receive assistance with activities of daily living and/or medical observation and care. HC services are assigned to patients by specially trained staff working outside HC services who determine the type and extent of care for which each patient is eligible. Patients who are admitted into Norwegian HC services are systematically assessed in terms of their care needs, based on the International Classification of Functioning, Disability and Health system developed by WHO (21, 22), resulting in an activities of daily living (ADL) score. The total score is the average of five sub-categories: (1) social function, (2) cognitive function, (3) ability to take care of their own health, (4) domestic responsibilities, and (5) self- care. Funding available to HC nursing services is allocated for each patient depending on their total ADL score. ADL scores are required to be updated at least twice a year by especially qualified staff (usually line managers).

ADL scores provide a comprehensive picture of patients' care needs. ADL scores allow for estimation of the time required to care for patients. They also allow for estimation of the physical demands associated with caring for patients. The feasibility study preceding the intervention that is evaluated here found that the self-care dimension was a sufficient proxy to measure levels of strain associated with the physical handling of patients (23). The self-care dimension was employed to classify patients in terms of how physically demanding it may be to care for them (19).

HC units employ a wide range of healthcare workers with varying qualifications, including registered nurses, nurse specialists, assistant nurses, disability nurses, occupational therapists, physiotherapists, social workers, and assistants. Patients vary in terms of both their age, care needs and the duration for which they receive care. Workloads and requirements regarding skills and competence fluctuate accordingly continuously and sometimes substantially due to, for example, new patients, deceased patients, admissions to higher levels of care, or changing care needs.

The study context comprises 12 geographically distinct HC units that are located in Trondheim, Norway. Each HC unit is composed of between two and four teams serving patients in a subdivision of geographic areas. Both HC units and teams accordingly differ in terms of geographic challenges such as distances and parking opportunities, and in terms of patient numbers and composition. All HC units except one are situated in an urban setting. One HC unit is situated in a more rural setting, which corresponds to a smaller size of the HC unit, potentially more time between assignments and longer driving distances.

Operations managers (OMs) plan work schedules for HC workers containing up to around 25 patient assignments per shift, depending on the length of individual assignments. In the planning process, they must consider many criteria such as patient needs, staffing availability, staff needs, efficiency targets, and continuity of care, to name only the most important. Work schedules specify which tasks are to be performed during each assignment, as well as the duration estimated for each assignment.

The HC services context is also characterized by ongoing changes pertaining to, for example, legal and regulatory frameworks, organizational changes, new procedures, and new technology. One that is important to mention is the implementation of a new electronic patient journal system (EPJ), which was introduced shortly before the planned intervention and caused an unpredicted sharp increase in the administrative resources required to run HC units. This led to delayed start of the intervention and a shortening of the intervention period.

### 2.2 Theoretical framework

Proctor et al. (24) have developed a comprehensive taxonomy designed towards an understanding of implementation processes and implementation outcomes. It is an established understanding that healthcare settings are complex, dynamic, and often unpredictable contexts (25). The choice to use Proctor et al.'s (24) implementation outcomes framework for this qualitative process analysis study is based on its clear alignment with the complexities of healthcare settings and its comprehensive approach to evaluating interventions in these contexts. Implementation outcomes are distinct from intervention outcomes (24, 26–28). Implementation outcomes result from deliberate and purposive actions to implement new ways of doing things. Implementation outcomes are precursory and conditional for “attaining subsequent desired changes in clinical outcomes or service outcomes” [(24), 66]. As such, understanding implementation outcomes enables an understanding of intervention outcomes. This qualitative process analysis study focuses on implementation outcomes. Thus, the framework provided by Proctor et al.'s (24) is an ideal choice for understanding how this intervention was integrated into practice and how it may eventually lead to meaningful service outcomes.

We will briefly outline the analytical dimensions for analyzing implementation outcomes from Proctor et al.'s (24) taxonomy that we included in our analysis. First, acceptability refers to “the perception among implementation stakeholders” (in this case OMs and HC workers) that an intervention “is agreeable, palatable, or satisfactory” [(24), 67]. What were OMs' and HC workers' initial thoughts about the GoldiCare intervention? Second, adoption refers to “the intention, initial decision, or action to try or employ an innovation or evidence-based practice” [(24), 69]. In the case of this intervention, this refers to OMs' adoption of the GoldiCare tool [see (19)]. Third, appropriateness is understood as “the perceived fit, relevance, or compatibility of the innovation or evidence-based practice for a given practice setting, provider, or consumer; and/or perceived fit of the innovation to address a particular issue or problem” [(24), 69]. Do OMs and HC workers think the GoldiCare intervention is a fitting way to address the goals of the intervention? Fourth, feasibility refers to “the extent to which a new treatment, or an innovation, can be successfully used or carried out within a given agency or setting” [(24), 69]. Do OMs and HC workers believe the intervention to be workable within the current HC services context? Fifth, fidelity refers to both the degree to which an intervention is implemented and whether it was implemented as prescribed. The analysis here supplements quantitative measures of fidelity [see (20)] and as such is focused on the latter, investigating the ways in which OMs employed the GoldiCare tool.

The dimensions penetration, sustainability, and cost were not included in the qualitative process analysis. Regarding penetration and sustainability, this was due to the early stage of the intervention, where one cannot expect an “integration of a practice within a service setting and its subsystems” [(24), 70]. Cost was excluded due to an ad priori condition that the intervention was to be cost neutral [see (19)].

## 3 Materials and methods

### 3.1 Data collection

Individual interviews and focus groups were conducted in HC units in the intervention arm. OMs were interviewed individually, since the focus here was to attain a comprehensive picture of their reasonings for and their ways of going about adopting the GoldiCare tool. Additionally, HC workers were interviewed in focus groups to gain insights into various understandings of and to promote discussions about issues pertaining to acceptability, appropriateness, and feasibility issues. In HC unit 6, only their OM was interviewed, as this unit withdrew from the intervention during its initial stage.

In a collaborative effort by all authors, semi-structured interview guides and focus group guides were developed (see Supplementary Materials). Both individual interviews with OMs and focus groups with HC workers were aimed at gaining insights into reactions, ideas, attitudes, beliefs, knowledge, and experiences pertaining to implementation outcomes as defined in the theoretical framework outlined earlier. Focus group guides did not contain questions pertaining to the use of the tool, since only OMs worked with the tool.

A semi-structured design allowed for the systematic coverage of analytical dimensions, in addition to further probing and exploration of potentially new themes. Some questions were adapted to the study's HC services and OMs were, for example, asked questions about the relevance of staff coverage, continuity of care, geographical teams, and patient-carer relationships. Due to an experience-based understanding of diverging ways of understanding physical demands and diverging workplace cultures around how to meet these demands, both interview guides and focus groups also contained questions relating to perceived support by the leadership.

### 3.2 Recruitment

All OMs in the intervention units were invited to participate and asked to provide active consent. They were recruited through established contacts with OMs already using the tool (Table 1). To be eligible for interviews, OMs needed to be working in their role for at least 20% of a full-time equivalent (FTE). All OMs who volunteered were interviewed. As Table 1 shows, at least one OM in each intervention HC unit was interviewed. One line manager in HC unit 2 was also interviewed, since she was largely responsible for employing the tool for a three-week period during the intervention. HC unit 6 opted out of the intervention since the OM was new in her role. She agreed to participate in the interview, providing insights on her understandings surrounding the intervention more generally, if not also on working with the tool. Since at least one OM from each intervention unit was interviewed and the saturation point was reached—with fewer new insights and no additional patterns emerging in the data—the ability to gather further new information had been achieved.

**Table 1:** Total number of operations managers (OMs) in each intervention home care (HC) unit and number of OMs interviewed in individual interviews in each HC unit.

HC unit	Total number of OMs employed in each HC unit	Number of OMs interviewed in each HC unit
1	3	2
2	6	2
3	3	2
4	2	1
5	2	2
6	1	1

All HC workers in the intervention units were invited to participate and asked to provide active consent. Recruitment for the focus groups took place via line managers. Inclusion criteria were that participants had been working as HC workers in their respective HC units during the intervention period in at least a 50% FTE position. As Table 2 shows, one focus group was conducted in each HC unit. Between three and four participants participated in each focus group. Recruitment of at least four participants was challenging and was not achieved in two units, since HC workers needed to be relieved from their normal work duties at the same time during a shift. These focus groups were nevertheless included in the analysis since we believe that overall variation of views was achieved not only within one HC unit, but across intervention HC units. In the same vein, topics not pertaining to the employment of tools were discussed during both focus groups and individual interviews, providing an additional variety of viewpoints. No focus group interview was conducted in HC unit 6, since this unit opted out of the intervention at the start of the intervention. Since at least one focus group interview was conducted with HC workers from each intervention unit and the saturation point was reached—marked by fewer new insights and no additional patterns emerging—the ability to gather further new information had been fulfilled.

**Table 2:** Total number of home care (HC) workers in each HC unit, total number of HC workers employed as at least 50% full-time equivalent (FTE) in each HC unit, and number of HC workers interviewed in focus group interviews.

HC unit	Total number of HC workers	Total number of HC workers in at least 50% FTE	Number of HC workers that participated in focus group interviews
1	95	59	4
2	67	39	3
3	76	54	3
4	79	80	4
5	24	24	4
6	67	36	0

### 3.3 Sample

As Table 3 shows, HC units varied in terms of number of employees, approximate number of patients assigned to each HC unit (approximate figures due to continuous fluctuation), how many OMs were employed and whether they were employed in that role on a full-time, as opposed to a part-time, basis.

**Table 3:** Home care (HC) units participating in the intervention and numbers of full-time OMs, part-time OMs, full-time employees, and patients.

HC unit	Full-time OMs	Part-time OMs (OMs working in their OM role 80% or less)	Number of employees with min. 50% position	Approx. number of patients	Number of teams
1	2	1	95	250	3
2	0	5	67	250	3
3	2	1	76	300	3
4	1	1	79	250	3
5	0	2	24	100	2
6	1	0	67	225	3

As Table 4 shows, a total of 25 participants took part in both individual interviews with OMs and focus groups interviews with both HC workers and OMs. It also shows interview and focus group participants in terms of age, gender, professional title, FTE, years worked in the HC sector, and their various roles in their HC units. The composition of participants is considered to be symbolically representative (29) of HC workers in the study context.

**Table 4:** Participants in terms of home care (HC) unit, age, gender, professional title, percentage of FTE position, number of years in the HC sector and professional role.

Participant	HC unit	Age	Gender	Professional title	FTE	Years worked in HC sector	Role in HC unit	Participant in individual interview	Participant in focus group
PD	1	34	F	AN	100	15	OM	Yes	No
FF	1	27	F	AN	100	3	HC worker	No	Yes
FL	1	28	M	OT	100	4	HC worker	No	Yes
PO	1	29	F	RDN	100	7	OM	Yes	No
FP	1	46	F	RN	100	3	HC worker	No	Yes
FY	1	25	F	OT	80	3	HC worker	No	Yes
PLI	2	44	F	RN	100	18	Leader	Yes	No
FK	2	41	F	RN	100	12	HC worker	No	Yes
FR	2	25	F	RN	100	2	HC worker	No	Yes
PFS	2	28	F	RDN	50/50	4	OM HC worker	Yes	No
PB	3	53	F	AN	100	25	OM	Yes	No
FG	3	25	F	AN	80	4	HC worker	No	Yes
PM	3	40	F	AN	100	12	OM	Yes	No
FN	3	24	F	RN	100	2	HC worker	No	Yes
FV	3	60	F	AN	60	25	HC worker	No	Yes
FA	4	47	F	AN	100	25	HC worker	No	Yes
FE	4	34	M	AN	100	6	HC worker	No	Yes
PJ	4	41	F	AN	100	15	OM	Yes	No
FQ	4	63	M	AN	100	18	HC worker	No	Yes
FT	4	28	F	RN	100	1	HC worker	No	Yes
PFC	5	22	F	AN	50/50	2	OM HC worker	Yes	Yes
FH	5	45	F	AN	100	16	HC worker	No	Yes
FW	5	38	F	AN	90	20	HC worker	No	Yes
PFX	5	30	F	AN	100	11	OM HC worker	Yes	Yes
PU	6	51	F	RN	100	3	OM	Yes	No

Explanation of abbreviations: F, female; M, male; RN, registered nurse; RDN, registered disability nurse; AN, assistant nurse; OT, occupational therapist.

### 3.4 Conducting interviews

Both individual interviews and focus groups were conducted at participants' workplaces between April and May 2023. Line managers provided rooms and freed participants of their regular work commitments. Individual interviews lasted between 45 min and an hour; focus groups lasted between an hour and 70 min. Author HF conducted all individual interviews with OMs. Focus groups were conducted by author HF and author SR together to ensure coverage of topics and probing, facilitate participation and ensure a balance of the emic and etic perspective (30). Author FKL had the main responsibility for conducting the intervention, and was therefore not involved in conducting the interviews.

### 3.5 Qualitative data analysis

All interviews were transcribed in full verbatim by both author HF and author TMG. Interviews were then analyzed through a three-step content analysis (31, 32); see also (33). As a first step, all authors read interviews and focus groups to obtain a sense of the whole, identifying broader dominant topics and sentiments. Involving all authors in the initial analysis strengthened validity and context-based reliability, in particular since it combined both the emic and etic perspectives of the various authors. This first analytical step was deductive in the sense that meanings associated with responses were derived from the dimensions of the theoretical framework. Some responses did not immediately fit into the existing analytical dimensions and were thus coded into preliminary remnant codes which at least initially were left outside the existing analytical dimensions. In the second step, the author HF went systematically through all interviews again from a more bottom-up approach, identifying meaning-bearing text units in broad relation to the dimensions of the analytical framework and developing a coding tree in correspondence with the dimensions in the theoretical framework. In the third step, initial coding results and efforts to organize existing and emerging themes were discussed and reorganized in several iterative cycles. Three group discussions with all authors took place, resulting in shared understandings and providing the basis for how the findings were presented here. The analytical process was aided in terms of its systematic nature by the qualitative analysis program NVivo 14 (Lumivero, Denver, CO, USA).

The voices of all participants were given equal weight in the analysis. However, the quotes selected to support the findings were chosen for their varying illustrative capacity and conciseness.

## 4 Results

In the following section we will describe implementation outcomes corresponding to the Proctor et al. (24) theoretical framework outlined in section 3. HC unit 6 opted out of the intervention at the beginning, so the perspectives of HC workers from this unit are not included.

### 4.1 Acceptability

Regardless of their role, participants described their initial reaction to the idea behind the intervention as positive, although OMs were all at least somewhat concerned about the potential additional work it might entail.

#### 4.1.1 Good idea

Participants welcomed the idea of a more even distribution of the workload due to “*staff needing variation when it comes to the tough work*”. *(PO)* They also described how they approved of the intervention's idea of rotating heavier and lighter work schedules. This participant argues, for example, that being assigned the same schedule for too many days in a row is “tough work” and that rotating these patients over the span of a week is a good idea because:

“if I get the same work schedule over time, I begin to feel that it is getting a bit tough. So, it's good to remain in your team, but that they rotate the work schedules a little more within the team” (FG).

#### 4.1.2 One more thing

OMs generally had initial concerns that the intervention would translate into extra work and that it was “*yet another thing we need to register*” *(PB)*. Some worried how to “*find the time to do this” (PO).* Their concerns were heightened since the intervention coincided with the implementation of a new electronic patient journal (EPJ) system. As this OM describes it:

“At the very start I kind of thought “oh no, not another thing!’, because of Helseplattformen (name of new EPJ)… which was a massive thing already, still is.” (PM)

### 4.2 Appropriateness

Appropriateness issues are divided into two themes: the time horizon and, the theme most extensively talked about; the self-care ADL not being a good proxy for physical demands.

#### 4.2.1 Appropriate time horizon

Referring to the weekly distribution of work schedules, OMs considered the weekly time horizon appropriate. They argued that it offered both sufficient potential for variation and the ability to respond to unforeseen changes, while not losing sight of the weekly target of distribution. PFS argues that it is “*good that it is a week and not more, because everything changes all the time”.* Especially for the smallest HC unit, having at least a week is described as the minimum time frame in terms of achieving sufficient variation between heavy and less heavy work schedules. An OM from the smallest HC unit argues that one week enables sufficient rotation, given that their HC unit operates with under ten work schedules and accordingly relatively few employees per shift compared to up to around 20 in the largest HC units.

“We are so few employees, that it is good this way, so that we can rotate enough where they are going” (PFC).

#### 4.2.2 Self-care ADL score does not sufficiently capture experience of physical demands

Some participants argue that the full ADL score (i.e., all five dimensions, including self-care ADL) may provide a good orientation for when patients may require more physically demanding care. However, no participant argues that the self-care ADL score on its own is a sufficient proxy for a “heavy” patient. Three issues underly this.

##### 4.2.2.1 ‛Skewing' sub-dimensions

One issue is that some of the subdimensions in the self-care ADL, such as the ability to eat or to get to places without assistance, lowers the overall self-care score, while caring for these patients is experienced as physically demanding. This discussion exemplifies this:

PFC: She doesn't have a high score. She manages to prepare her own food. She is sociable and gets herself from A to B.

FW: But it's her we are wearing ourselves out on.

PFX: Yes, it's her we are wearing ourselves out on.

Participants also frequently talk about how aspects not captured by patients' self-care ADL score make the experience of caring for these patients more demanding. This OM describes how care for patients and the level of demands experienced while doing so are both contextualized and subjective.

“When we talk about heavy patients, we don’t talk about their ADL score. We talk about the experience of being in their home” (PJ).

Psychosocial demands are mentioned frequently and considered an important part of the overall experience of demands. However, since our analysis focuses on understanding implementation processes pertaining to an intervention aimed at redistributing physical demands, we remain focused on issues pertaining to the experience of physical demands. Two themes emerged in this regard: the importance of the physical care environment and the quality of the patient-carer relationship.

##### 4.2.2.2 Physical environment

Participants considered the physical environment important for understanding the overall experience of physical demands. The overall care experience in terms of physical demands can be either positively or negatively impacted by stairs, narrow spaces or other physical barriers for ergonomic patient handling, or using equipment for doing so. This OM describes this when talking about two patients for whom the self-care ADL did not reflect physical demands experienced during care, due to a physical care environment that either compensated or made care unnecessarily demanding.

“With patient X we had to be two, but we could have been one really, because everything was optimized. She did very little herself and had a high ADL. Then we have patient Y where we need to be two. He manages quite a lot, but everything is hard work because of where he lives. Small and narrow, with stairs, door frames. (…) Otherwise, one person could have done that” (PJ).

##### 4.2.2.3 Patient-carer relationship

Participants also commonly mentioned how the level of cooperation and mutual understanding that is fostered within a good patient-carer relationship is considered important for how easy it is, also physically, to perform care work. This issue was typically mentioned when describing additional physical demands. Participants in all HC units described how a lack of cooperation could affect the amount of handling required, the type of handling required, whether care aid equipment was available, and whether such equipment could be employed as intended. This HC worker, for example, explains how it is physically more demanding to care for patients who do not cooperate around ergonomic principles.

“Patients often have their own ideas about how they want things done. (…) Then you end up doing things in a way that are not really good for you, all because they get to decide” (FY).

### 4.3 Feasibility

Participants described feasibility issues that fall into two categories. OMs described challenges with regard to the use of the GoldiCare tool. Both HC workers and OMs described issues pertaining to the ability to rotate work schedules in alignment with the intervention.

#### 4.3.1 Cumbersome

While OMs argued that the tool was easy to understand, and that technical support was both accessible and helpful, they also found the tool resource to be demanding, as they needed to work with the tool parallel to their existing EPJ. Some therefore suggested that integrating the tool into their existing EPJs would overcome these issues. This OM describes this as follows:

“I wish we could just get everything directly from Helseplattformen (…). Then you wouldn’t have to sit and go through all the names, fill in names… Yes, because that was really cumbersome” (PJ).

#### 4.3.2 Adding mental load

Planning of work schedules takes place in a very complex and dynamic context. OMs need to consider many, continuously changing, factors in their planning. Fluctuating patient numbers and care needs, staff being on partial or full sick leave, various types and levels of qualification and experience, minimizing driving time, or aiming for continuity of care are examples, illustrating how planning of work schedules is a mentally demanding process, coined by one OM as an “*ongoing and incomplete puzzle*” *(PD).* GoldiCare adds another layer of complexity that increases the mental load and acts as a barrier to adoption of the tool. This OM summarizes why adoption of the tool was not successful in their unit as follows:

“I understand the idea behind it, but everything is too much in flux. (…) When we plan for the next day, there will be changes. Sick leave, patients being admitted, formal qualification—who can take over for someone who is sick, those kinds of things. (…) So everything that happens is prioritized over GoldiCare” (PFS).

#### 4.3.3 Challenges to the variation of work schedules

Participants named three issues when explaining why rotating work schedules in order to achieve a weekly balance was challenging: (1) the importance of continuity of care, (2) the organization of work within geographically based teams, and (3) the availability of staff in terms of both numbers and qualification levels.

##### 4.3.3.1 Continuity of care

Resistance to the variation of work schedules among both OMs and HC workers is rooted in ideas of how continuity of care improves quality of care and minimizes strain for both patients and staff. Although staff may welcome some variation, if there is too much of it staff may struggle to manage “*everything that you were supposed to do” (FG)* or manage only “*a half-bad job” (FN)*. Being assigned unfamiliar work schedules is also described as “*exhausting” (FG)* and increasing the “*mental load” (FT).*

Accordingly, in all HC units, both OMs and HC workers perceived continuity of care as an issue that made variation challenging. The importance of continuity is summarized by this OM:

“For both patients’ and our sake, we think about continuity. (…) And staff, they want to go to their own patients (…)” (PFS).

In some units, the political directive makes continuity an even more salient issue. This OM describes how both the leadership and OMs seek to prioritize continuity, due to the political directive around continuity of care.

“The intervention’s set-up doesn’t fit in with how we are supposed to work. It crashes. (…) We are measured based on continuity, and we can’t achieve continuity if we are to use the tool as intended (…)” (PJ).

##### 4.3.3.2 Geographically based teams

The organization of work into geographically based teams, which is rooted in ideas about the importance of continuity of care, a political directive towards ensuring continuity, and economic considerations related to minimizing transport time, is perceived as a challenge for achieving sufficient rotation. HC workers generally stress the importance of working within their teams and caring for patients they know. Rotation within their teams is thus not considered as problematic as rotation in between teams.

“We are grouped into three areas, each with a number of lists, some heavier than others, so that you can still rotate within that area” (FT).

In some HC units, geographic distances between teams make economic barriers a particularly salient issue in this context.

“To give somebody from the X team a patient from the Y team is tricky, because then you need to drive from X to Y, and that takes maybe 20 min” (PFC).

When “heavy” patients happen to be clustered in certain teams the salience of team-based care becomes particularly obvious. This OM describes how this was a particular challenge in their unit.

“The patients with high scores were all assigned to the same team. So, I had to send the same staff there, really. It wouldn’t have been good to send someone there that had never been there before, just so that they skipped a heavy work schedule” (PO).

##### 4.3.3.3 Availability of staff

OMs described how they needed to accommodate resistance to rotation among staff. This OM describes how her initial attempts to rotate work schedules were met with resistance by HC workers.

“We tried to rotate in the beginning. (…) We tried to explain, because they [HC workers] asked what this is all for and so on. But many wanted to keep their lists regardless” (PJ).

OMs explained how they avoided too much rotation, and in particular rotation between teams, to avoid risking negative HC worker reactions, decreased job satisfaction, or even sick leave. This OM argues how she was careful to minimize rotation to unfamiliar work schedules, with both staff satisfaction and potential sick leave in mind.

“If I send staff to totally new lists, it can lead to uncertainty and cause staff to get stressed. They may not even turn up the next day” (PM).

Staff considerations are seen as a barrier to rotation in ways related to both continuity of care and geographically based teams. In addition, sending the right staff (in terms of both formal and experienced-based expertise) at the right time to the right patient is considered crucial for quality of care. While this was mentioned by many, this issue was particularly pronounced as a barrier to rotation in the smallest HC unit. As one of the OMs there explains, fewer staff and fewer highly qualified staff meant that “*achieving a balance can be difficult, because it depends on who I need on what list.”* (PFX). When patient needs or numbers change, or when staff are absent, more qualified staff may need to be assigned fuller and thus heavier work schedules. As this RN puts it:

“If there is only one nurse, she will have to do more assignments” (FH).

While this challenge may be less pronounced in larger units, since a larger pool of staff makes rotating work schedules within teams potentially more feasible, a pronounced focus on specialization may counteract this. Formally more qualified staff, such as registered nurses, may specialize in tasks that are physically less demanding such as medical procedures, clinical evaluations, medicine room responsibilities, and administrative tasks.

“Nurses go often pure nurse lists, and they tend to be less physically demanding” (FR).

### 4.4 Adoption

A small number of OMs decided not to take part in the intervention. Reasons for this were only established in the case of two OMs (PD and PU), who nonetheless volunteered to be interviewed. PD's decision was based on not considering the self-care ADL a good enough way of defining “heavy” patients as well as concerns about the potential added workload associated with the intervention. PU explained that because she was new in her role, she did not want to commit to the extra work in conjunction with the intervention. Among those who took part in the intervention, leadership support, time to focus, and teamwork/cooperation were provided as factors affecting their adoption of the GoldiCare tool.

#### 4.4.1 Leadership support

Leadership support matters, both in terms of explicit “moral” support and in terms of providing resources (the latter evident as terms of time/focus being relevant for adoption, as described below). In some units, the leadership was not explicitly mentioned as an issue. However, OMs in these units did not describe struggling to find time or ability to focus on working with the tool, therefore making leadership likely to be less relevant. In contrast, in HC units, where lack of time/focus were reported more emphatically as a barrier to adoption, leadership support was also a bigger issue. This OM describes how the leadership evolved from being somewhat passive towards displaying a more negative attitude towards the intervention due to concerns around feasibility.

“Nobody asked “how is it going? Does it take a lot of time? (…) And when they realized that we should not focus on continuity, they became much more negative’” (PJ).

Another OM describes how there was a sense of expectation, while OMs also struggled to find time to employ the tool.

“It was just something the leadership had said yes to us doing, and then it was us having to do the work. Then you develop this attitude of ‘why should I?’” (PFS).

In response to the OMs asking for more resources, the leadership became actively involved in using the tool to investigate these claims and eventually provided more resources. However, a negative attitude among OMs towards the intervention remained, partly due to continued feasibility and appropriateness issues and partly because additional resources were only provided after further investigation.

“We had to ask for time for something they asked us to do. Had they just given it to us in the first place, I would have had a better feeling about it” (PFS).

In another unit, part-time OMs who struggled to find the time/focus to adopt the tool were promptly allocated more time, only for this extra time to be taken up by more OM work associated with a sudden increase in patient load, and additionally, these OMs needing to be reallocated to work with patients.

“We got more time but then had to go out on lists anyway. There were a lot of unforeseeable things that happened, when we had plans to work with GoldiCare” (PFC).

#### 4.4.2 Time to focus

As shown in Table 3, HC units vary substantially in terms of OM resources and how these are organized. Findings suggest that sufficient time and thereby ability to focus affect OMs' ability and motivation to adopt the tool. In HC units with two full-time OMs, OMs described fewer challenges when adopting the tool and more easily found ways of addressing potential challenges. Working in a team with other OMs may facilitate sufficient focus to work with the tool. In contrast, OMs from HC units where they work either by themselves or only part-time describe greater challenges with regard to finding the necessary time and focus required to adopt the intervention. How much OMs talk about time and resources affecting the adoption of the tool differs accordingly. In units with two full-time OMs, time pressure is not mentioned as a barrier. In other HC units, however, OMs describe how a high workload prevents them from engaging more with the tool, using words and phrases like “*difficult”* (PFC), “*not enough time”* (PFX), “*time-consuming”* (PFS) “*overwhelming”,* and “*not a chance”* (PJ).

#### 4.4.3 Teamwork

A well-functioning team of OMs promotes adoption of the tool as it allows for sufficient task specialization, operative support, and developing a shared strategy for adopting. In HC unit 3, for example, OMs had established good routines and considered themselves a good team, facilitating the development of daily routines conducive to adopting the tool.

“We both have been OMs for three years now. (…) We have found a good distribution of tasks between us. (…) And we talk with each other. We have a good dialogue between us to manage what we are set out to get done each day” (PM).

In contrast, when OM resources are fractioned into small roles, OMs may not develop a cohesive approach for adopting the tool, as explained by this OM:

“Some are a little more like ‘Yup, we can do this!,’ while others are a little more like ‘No, I’m not going to start investing time in this!’ Some barely know what GoldiCare is about, kind of…” (PFS).

This OM explains that while she and another OM had the main responsibility for ensuring the tool was adopted, they struggled to get other OMs to engage with the tool, and the tool was consequently not adopted as intended.

“We filled it in. … But we are in the office two, three times a week. The two who sit in here mainly, they were not interested” (PFS).

### 4.5 Fidelity

Fidelity was affected in two main ways: first, the degree of incorporation into the planning of work schedules and thus the degree to which work schedules were rotated with the previous days in mind; and second, defining “heavy” in other ways than prescribed in the intervention protocol.

#### 4.5.1 Varying incorporation

There was variation in terms of how proactively OMs incorporated working with the tool when planning work schedules. Some OMs tried to balance the workload using the tool and explored ways of integrating this into their established planning work. This OM describes how she developed a routine whereby first thing in the morning she used information about the previous days to plan subsequent days, to achieve a more balanced weekly distribution.

“I see where they went and if they have been a lot to those with a high ADL, on the heavy work schedules, I gave them lighter lists” (PO).

In contrast, others struggled with filling in the tool altogether, and when they did, only registered the status quo without using this information to redistribute subsequent days.

“We managed to register it, but what we did with it is another question” (PFS).

Others again described how they did not fill in the tool consistently but maintained focus on the underlying principle of balancing the weekly workload.

“We made sure staff didn’t get the same heavy work schedule on every shift. (…) But in terms of the tool—that was way back there [pointing at back of the head]” (PJ).

#### 4.5.2 GoldiCare “plus”

Among those who employed the tool, all did so with a more holistic definition of “heavy” than in the intervention. We coined this approach “GoldiCare plus”. As described in the section on appropriateness, OMs struggled with what they perceived to be too narrow a definition of “heavy”. This OM, for example, describes how this more holistic approach reflected their attempt “*to use the square box that you gave us to work with.”* (PJ).

In all HC units, some patients with a lower self-care ADL score than the cut-off point, but who were deemed as “heavy” patients regardless, were included in the list of “heavy” patients, which in turn was the basis for classifying work schedules as “heavy”, “medium heavy”, and “light”, respectively within the intervention logic. As this OM describes it:

“When we plan lists it’s about ADL yes, but also about those patients with a low ADL, but where we know they are ‘heavy’” (PO).

## 5 Discussion

The overarching goal of this qualitative study was to better understand why and how the GoldiCare intervention was implemented as it was. Employing Proctor et al.'s (24) theoretical framework of implementation outcomes, this study was a component of the process analysis of the GoldiCare intervention [see (19)], complementing the quantitative evaluation of both fidelity and reach [see (20)].

Findings resulted in insights into (1) barriers to and facilitators for implementing the GoldiCare intervention, (2) underlying processes behind the reach of the intervention, (3) understandings of adaptations to the intervention and thus fidelity, and (4), based on the previous three insights, outlining future challenges and solutions for GoldiCare interventions in the Norwegian HC sector.

The GoldiCare intervention was welcomed in terms of its fundamental idea of balancing the workload of HC workers in order to rebalance physical demands and in this broad sense was deemed acceptable by HC workers (see the earlier definition of acceptability and see Proctor et al.'s [(24)] framework). However, quantitative process evaluation showed that a number of units did not implement the intervention as intended in terms of intervention outcomes' fidelity and reach [see (20)]. Indeed, one of the HC units withdrew from the intervention. This withdrawal was due to a new staff member starting as OM, who at the time felt not capable of taking on extra responsibilities associated with the intervention in addition to being trained in her new role. HC workers in this unit were consequently not interviewed, since the current process evaluation focuses on intervention outcomes, which presume participation in the implementation.

This study provides a qualitative supplementation of the process evaluation and helps to identify several implementation outcomes. As described in the results section, several issues pertaining to appropriateness, feasibility, and adoption [see section on theoretical framework and Proctor et al. (24)] provide a better understanding of the moderate fidelity and reach of the intervention.

We argue that these issues can be further synthesized into four core issues that are interwoven with several of the analytical dimensions of the theoretical framework: (a) the need for a more holistic and experience-based way of measuring physical demands, (b) complexity and unpredictability, (c) organization-level issues, and (d) operational autonomy.

### 5.1 The need for a more holistic and experience-based way of measuring physical demands

Issues surrounding how to define and measure physical strain are primarily an appropriateness issue, but indirectly also relevant for feasibility and adoption. Despite the feasibility analysis preceding the intervention suggesting that the self-care ADL score can be considered a sufficient proxy for physical demands (23), our findings suggest that the self-care ADL score may not sufficiently encompass HC workers' experience of physical demands and may thus not be seen as an appropriate basis for distributing the workload. While participants tend to describe physically “heavy” patients in terms of “just knowing” or the “overall experience”, probing resulted in identifying several issues that may help close the gap between HC workers' experiences and a proxy for physically “heavy”.

One issue is that some subcategories in the self-care ADL score may dilute proxy strength. Higher levels of functioning related to eating and mobility outside the home are included alongside dimensions that directly affect physical demands for HC workers, such as patients’ ability to wash themselves, go to the toilet, and move in and out of bed. These dimensions skew the overall score towards a “lighter” score, potentially resulting in patients falling outside the intervention's definition of a heavy patient, even though they may be experienced as “heavy” to care for.

Moreover, issues not captured in the self-care ADL score may affect the experience of physical demands. HC workers describe their experience of physically “heavy” patients' or, indeed, “heavy” assignments in contextualized, holistic, and relational ways. The physical care environment, the patient-carer relationship, and levels of cooperation between carer and patient are all relevant for HC workers' ability to work in accordance with ergonomic principles and thus may affect physical demands. Importantly, these issues are potentially interconnected. For example, the physical care environment may be affected by patients not being cooperative in terms of getting equipment, or refusing changes to their homes that facilitate a care-friendly environment. In addition to socioculturally based expectations on the side of patients and HC workers, regulatory and political issues may play a role in this. Ultimately, HC workers must negotiate expectations around patients' ability to maintain a non-institutionalized environment on the one hand, and their needs around working according to ergonomic principles on the other hand.

These findings are in line with Grasmo et al. (34), who found that HC workers operate at the crossroads between often competing mechanisms and motivations related to the regulatory environment, workplace cultures, physical care environments, patient-carer relationship, and economic drivers, all of which place responsibilities on HC workers' to minimize their physical demands, while navigating these potentially competing considerations. Others have also found that HC workers’ experience of physical demands is complex and interwoven with potentially less tangible aspects such as psychosocial stressors. For example, Miranda et al. (35) and Miranda et al. (36) found that patient violence affects musculoskeletal pain levels experienced by HC workers in American nursing homes. Overall, we therefore argue that “heavy” ought to be understood, captured, and targeted at the level of the care experience in a patient's home, as opposed to being treated as a set of attributes belonging to a patient independent of relationships and context.

The potential interconnectedness between the various implementation outcomes, as already described by Proctor et al. (24), is evident here. Proxy issues affect both willingness and ability to adopt the intervention according to the program logic. Proxy issues have resulted in a lack of engagement with the intervention among some OMs (reducing both adoption and fidelity). Not being convinced that the intervention redistributes “heavy” care work as experienced by HC workers, OMs did not always engage with the tool as intended (affecting adoption). OMs were also not as inclined to rotate work schedules as prescribed in the programme logic (affecting fidelity). OMs, when employing the tool, also developed what we coined a “GoldiCare-plus” approach, incorporating both their own and other staff's understandings of “heavy” patients or, indeed, “heavy” assignments (affecting fidelity). However, we argue that in making the intervention more appropriate from OMs' point of view, the GoldiCare-plus approach likely increased adoption of the intervention.

### 5.2 Complexity and unpredictability

It is a well-established idea that healthcare organizations are multi-layered, complex, and somewhat unpredictable environments [e.g., (25)]. This is also true of HC services. Myriads of considerations related to patients and their care needs; availability of staff in terms of numbers of qualifications and experience; allocating the right staff to the right patient at the right time; and geographically based care teams due to both economic targets and political goals around continuity of care, to name but a few, need to be taken into account when planning work schedules. Moreover, work schedules are always potentially in flux and may need adjusting due to sudden developments related to, for example, sick leave; increased care needs; patients being admitted to hospital; and staff swapping assignments for various reasons, to name but a few. This creates a substantial mental load for OMs associated with the planning of work schedules.

The GoldiCare intervention added another layer of complexity and as such affected both feasibility and adoption, albeit to various degrees and at various times across the participating HC units. Some OMs opted out at the beginning of the intervention. Others were able and willing to continue to engage with the intervention, but sometimes had to prioritize day-to-day operations instead of engaging with the intervention. Comparable effects have been shown for other interventions conducted in healthcare settings. For example, Czuba et al. (15), exploring risk factors leading to injury and increased turnover in home health aides, similarly found that unexpected changes on the side of both patients and staff reduced fidelity and thus affected projected outcomes of that intervention.

### 5.3 Organization-level issues

While HC units operate under the same overarching legislative, political, economic, and sociocultural frameworks, these overarching issues play out differently in each HC unit and, accordingly, affect feasibility, adoption, and fidelity in different ways. Our findings suggest that OMs, leaders, and HC workers in the participating HC units have varying understandings of how care can be and ought to be delivered. These varying understandings—when not aligned with the intervention (i.e., when appropriateness and feasibility issues arose)—may become visible in terms of adoption and fidelity issues. Explicit and implicit support from both leaders and colleagues affect OMs’ perceived ability and motivation to engage with the intervention according to protocol. Findings suggest, for example, that if the leadership expresses negative attitudes towards the intervention, because they perceive it to counteract continuity of care, OMs will be less engaged with the intervention. Findings also suggest that OMs are aware of the potential to incur resistance from colleagues or even contribute to higher sick leave rates if they rotate work schedules in ways that are perceived as producing more strain. This also serves as an example of the complex interactions between appropriateness issues, feasibility issues, adoption, and fidelity.

Findings suggest that leaders in HC units generally tacitly support the intervention. Some were more active, in terms of both showing an interest in the intervention and providing resources (mainly time for OMs to engage with the intervention). Leadership support in the sense of endorsement is important for OMs' engagement with the intervention. An example where this was challenged was when the leadership in one organization reacted negatively upon realizing that the intervention potentially counteracted the continuity of care principle. The latter is manifested in a political directive toward a target of a maximum number of HC workers visiting patients (37), while continuity of care is nevertheless not interpreted and enacted similarly across all participating HC units. It is also a sociocultural norm in Norwegian HC settings. Care ideologies and care cultures are important for understanding certain practices around how care is given (38, 39).

However, the organization-level perspective is relevant here in that HC units differ in terms of how those working there, as leaders, OMs, and HC workers more generally, translate this norm and political directive into practice. Although the intervention's inherent focus on increased rotation conflicts with the principle of continuity of care and thus potentially represents feasibility issues hindering adoption and affecting fidelity, this was not a salient issue in all HC units. Similarly, other broader frames for action such as the scarcity of funding, recruitment and staffing issues, and so on, affect perceptions around appropriateness and feasibility differently in different HC units. An example is how OM resources, in terms of time to engage with the intervention, do not necessarily translate into increased adoption and fidelity. In some units, OMs' lack of sense of ownership regarding the intervention negatively affected their engagement with the intervention. Established teamwork between OMs around the intervention also matters for engagement with the intervention and incorporation of the tool into the planning of work schedules. This may explain why there was no simple and tight relationship between time available to OMs on the one hand and adoption of the intervention as intended in the protocol on the other (adoption and fidelity in other words).

These findings suggest that while some of the differences in adoption and fidelity can be explained by tacit factors such as availability of resources or the size of the organization, differences may also be based on diverging beliefs of HC workers and established practices around how to give care and how to organize care. HC units differ in terms of how they distribute tasks between staff, how they organize patient-related work in teams, and how decision-making processes are delegated from leaders to OMs, and so on. This variation affects feasibility, appropriateness, adoption, and fidelity issues and, importantly, perceptions around these issues, in various ways. As such, it affects the implementation of the GoldiCare intervention. In the same vein, Eide et al. (40), on analyzing facilitators and barriers for an intervention attempting to transform HC services towards being trust-based service provision systems, argued that actors and groups of actors within HC units may produce diverging implementation outcomes based on divergent interpretations of both laws and regulations and how these, in turn, affect actions relevant for implementation.

### 5.4 Operational autonomy

Our analysis suggests that operational autonomy is important to understanding implementation outcomes. Autonomy matters in terms of OMs' fidelity to the intervention protocol. OMs exercise autonomy when employing a GoldiCare-plus definition of “heavy” patients, knowing that adhering strictly to the intervention's definition of “heavy” would counteract goals towards good quality care (based on continuity of care), as well as less demanding care work. Based on a similar rationale, HC workers may act autonomously when swapping assignments and thus ultimately affecting intervention outcomes. Only in one HC unit was this practice discouraged, whereas it was generally considered acceptable in other HC units (which also highlights the importance of organizationally based cultures of care, as discussed earlier).

Acceptance may be based on the idea that HC workers need to navigate care work at the interspaces between patients, staff, HC organizations and wider frameworks (34, 38). Autonomy is also considered an important dimension of both HC workers' identities centered around “good” care (41, 42) and good (self-) leadership in healthcare contexts more generally (31). In a sector already characterized by increased tendencies towards the monitoring and operationalization of tasks—resulting in a transformation from patient-carer relations to client-service provider relations (43, 44)—retaining a degree of operational autonomy allowing HC workers to care for whom they know best, and how they and patients see fit, is likely to reduce strain and sick leave rates (45), increase work satisfaction (34, 46, 47) and increase retention and reduce staff shortages (46, 47). Thus, accepting autonomy in this sense of operational self-leadership ought to be considered a necessary and an inherent aspect of work in HC settings (48). An intervention such as GoldiCare designed to reduce strain while maintaining productivity and quality, certainly ought to pay attention to potential adverse effects of reducing HC workers' autonomy.

### 5.5 Suggestions for future research and practical implications

First, we argue that GoldiCare interventions ought to rely on a proxy for not just “heavy” patients but also “heavy” assignments. Patient attributes, the physical care context, and the patient-carer relationship should be integrated. A proxy that captures the experience of a “heavy” assignment would reduce appropriateness issues and thus promote successful implementation and the achievement of intervention outcomes. However, generating necessary data for such a proxy might be challenging. Some data, such as patient weight, are already found in existing EPJs. Other data, such as patients’ physical living situations, patient-carer cooperation, and psychosocial aspects of the care experience need to be generated through assessments or evaluations. Not least, the generation of such data may be resource-intensive and potentially fraught with ethical or legal challenges. Second, we argue that GoldiCare interventions need to be feasible in terms of the complex and unpredictable context of the Norwegian HC sector. They need to be responsive to economic constraints, political and regulatory frameworks, and organization-level variations related to patient numbers and patient needs, staffing levels, geography, and so on. In other words, GoldiCare interventions need to be responsive across both place and time. Third, GoldiCare interventions need to accommodate sufficient autonomy for HC workers who engage directly with patients. This is important to avoid risking adverse effects regarding both quality of care and strain among HC workers.

We have two main suggestions for how future GoldiCare interventions can address these four main challenges. First, we propose better integration of a GoldiCare tool with EPJs to reduce mental load and facilitate the continuous integration of information pertaining to HC workers' experience of “heavy” assignments. Second, GoldiCare interventions need to accommodate continuous participation by HC workers, with the inherent goal of redeveloping the intervention to match the context in terms of both place and time.

Regarding the first suggestion, better integration with EPJs could reduce barriers in terms of lack of time and mental load for OMs. This could also reduce validity issues important for evaluating intervention outcomes, since proxy data that needs to be added manually could be minimized. Similarly, unplanned changes to work schedules due to swapping of assignments could be registered automatically, thereby eliminating the need for retrograde adjustment. Integration with real-time feedback from HC workers working with patients might also be a necessary and desirable strategy. Such integration could provide information on the care experience. A variety of indicators of experienced strain, ranging from key vital measurements from HC workers or quick-to-enter feedback data from HC workers pertaining to the care experience, could be of potential interest here. Operational autonomy may be supported, but also diminished, by such integration. The former, for example, since swapping might be less of a challenge, as integration removes the need for OMs to manually check previous work schedules. On the other hand, there is a risk that aspects of the care experience not captured by new indicators to measure strain would become invalidated and that HC workers' ability to choose whom to care for and how to do so might be curtailed.

Regarding the second suggestion, it must be emphasized that the GoldiCare intervention was participatory since its development incorporated feedback from stakeholders and HC workers (49). The issues raised in the focus groups suggest that the participatory process may not have been adequately led or sufficiently inclusive. While our findings may provide useful insights for redesigning future attempts to implement the GoldiCare tool and contribute to more successful implementation, we believe that the highly complex and dynamic context may call for a more continuous participatory approach than the one used in our study. It is possible that key perspectives were overlooked during the tool's development, or that variations in organization and culture between units may have played a role. One size is unlikely to fit all and unlikely to remain fitting over time. We believe that a more continuous and systematic approach to facilitating and promoting participation by those enacting the implementation of the GoldiCare tool is key to implementation. This is in line with von Thiele et al. (50), who argue for a more continuous and participatory approach to developing successful interventions. As already mentioned in the context of better integration of EPJs and ways of measuring how strenuous the experience of care is, our findings provide examples of the usefulness and necessity of a participatory approach. On the OMs' side, an obvious example of the usefulness of a participatory approach is evident in terms of OMs mitigating appropriateness issues and promoting adoption by compromising fidelity to the programme logic and developing a “GoldiCare-plus” approach. In short, although representing non-fidelity, the “GoldiCare-plus” approach makes the intervention work. Another example of the usefulness of a participatory approach is that participants suggested integration of the tool with EPJs, thereby rendering it more appropriate and feasible. This need for participation is further heightened when considering the highly contextualized nature of Norwegian HC contexts. Political directives, economic resources, organizational forms, and workplace cultures, to name but a few, all differ between HC units in the study context, and more so across Norway as a whole.

### 5.6 Strengths and limitations

One of the primary strengths is the high number of respondents, which provides a robust dataset that enhances the reliability and transferability of the results. A diverse group of respondents, including HC workers, as well as OMs with different perspectives, contributes to a rich, multi-faceted understanding of the topic. This diversity enables the research to capture a wide array of experiences and opinions, reflecting the realities of different stakeholders within the organization. Another important strength is the qualitative approach utilized in the study. This approach allows for a deeper exploration of the participants' attitudes, motivations, and personal experiences, providing rich, contextualized insights. These insights help us to understand not only what has happened, but also why certain trends or behaviors are observed during the implementation of the GoldiCare intervention. Additionally, the data collection and analysis were conducted by a multidisciplinary team, following a clear strategy to maintain a balance between the emic and etic perspectives, fostering reflexivity throughout the process. Moreover, the use of method triangulation is a significant asset. By combining qualitative data from both individual and focus groups with findings from the quantitative data, the research gains a more comprehensive view of the situation. The qualitative findings are supported and clarified by the quantitative data, making it easier to interpret the underlying dynamics and reinforcing the credibility of the conclusions.

Despite its strengths, the research does have some limitations. One potential issue is selection bias. Since those who are more motivated or interested in the topic may be more likely to participate, the sample might overrepresent certain viewpoints. This could limit the breadth of the findings, as the experiences and opinions of less motivated individuals might be underrepresented. Additionally, there is the possibility that only those who felt empowered or comfortable using their voice participated. This creates a risk of excluding perspectives from individuals who, due to fear, apathy, or other reasons, might refrain from contributing. Such bias could skew the results towards more outspoken or engaged respondents, thus limiting the study's ability to fully represent the broader population's views.

As a consequence of choosing Proctor et al.'s (24) implementation outcomes framework, one limitation is that the framework primarily focuses on early-stage implementation, which may not fully capture long-term integration or the sustained impact of the intervention over time. Additionally, the framework's emphasis on subjective perceptions of stakeholders, such as acceptability and appropriateness, may introduce bias, as these perceptions do not always align with objective measures of effectiveness. Furthermore, while the framework provides a structured approach to assessing implementation, it may not adequately address the broader contextual factors, such as organizational culture or external influences, that could also impact the success or failure of the intervention.

## 6 Conclusion

In conclusion, this qualitative study offers valuable insights into the implementation of the GoldiCare intervention, highlighting the barriers and facilitators that affected its implementation outcomes, including acceptability, appropriateness, feasibility and adoption within the Norwegian HC sector. Key challenges identified include the need for a more comprehensive and experience-based measure of physical demands, the competing priorities and unpredictability within HC settings, organizational-level differences, and the importance of providing operational autonomy to both OMs and HC workers.

The Goldilocks work principle holds theoretical potential to promote a healthier distribution of workloads among HC workers. Looking ahead, the findings from this study emphasize that successful implementation of GoldiCare requires further adaptation to the local context, acknowledging the dynamic and complex nature of HC environments. Additionally, technological development and integration with existing electronic patient journals (EPJ) would offer OMs greater insight into a broader range of factors impacting workload, enabling real-time adjustments to work schedules in response to fluctuating conditions.

Ongoing participatory approaches are recommended to maintain stakeholder engagement, ensuring the intervention remains contextually relevant and adaptable over time.

## Data Availability

The data that support the findings of this study are available from the first author (HF, hef@lukas.vgs.no), upon reasonable request.

## Footnotes

^1^The GoldiCare intervention was registered as a clinical trial on 08/05/2022 under NCT05487027.
