# Supplementary material for: “It's a good idea, but…”: a qualitative evaluation of the GoldiCare intervention in Norwegian home care services
Source: Front Health Serv. 2025 Jan 20;4:1511772. doi: 10.3389/frhs.2024.1511772 (PMC11789199; doi:10.3389/frhs.2024.1511772)
Supplement: Supplementary file 1 [file Supplementaryfile1.pdf]

Interview Guide for Focus Group Interviews with Home Care Workers (HC Workers)

ACCEPTABILITY

What were your first thoughts and feelings about the intervention?

APPROPRIATENESS

What do you think about the intervention now?

- Regarding the "just right" distribution of physical workload?
- Regarding balancing lists on a weekly basis?
- Regarding the intervention's potential to promote the health of employees?
- Other things?

What are your thoughts on using ADL/self-care as a basis for distributing lists?

- Are there additional ADL categories/other aspects that should have been included?

What are your thoughts on possible alternatives for distributing the workload so that the work becomes more health-promoting?

FEASIBILITY

How did you feel the intervention fitted into the daily work routine in your home care unit?

- In terms of professional coverage
- In terms of continuity
- In terms of relational factors (between patient and employee)
- In terms of geography

How were the needs of patients taken care of while the intervention was in progress?

How was the unit's productivity affected while the intervention was in progress?

How could the intervention possibly have been changed to better fit the daily work routine in your home care unit?

How do you experience the culture of change at your workplace?

What is needed for such an intervention to be used in home care in the future?
