# Supplementary material for: “It's a good idea, but…”: a qualitative evaluation of the GoldiCare intervention in Norwegian home care services
Source: Front Health Serv. 2025 Jan 20;4:1511772. doi: 10.3389/frhs.2024.1511772 (PMC11789199; doi:10.3389/frhs.2024.1511772)
Supplement: Supplementary file 2 [file Supplementaryfile2.pdf]

Interview Guide for Individual Interviews with Operations Managers (OMs)

ACCEPTABILITY
What were your initial thoughts and feelings about the intervention?

APPROPRIATENESS
What do you think about the intervention now?

- In terms of a “just right” distribution of physical work burden?
- In terms of balancing lists on a weekly basis?
- In terms of the intervention’s potential to promote employees’ health?
- Any other thoughts?

What do you think about using ADL (Activities of Daily Living)/self-care as a basis for distributing lists?

- Are there additional ADL categories/other aspects that should have been included?

What are your thoughts on possible alternatives for distributing the workload in a way that makes the work more health-promoting?

How did you experience using the tool to carry out the intervention?

- In terms of productivity
- In terms of your daily work

How did you find the intervention fitted into the daily operations of your home care unit?

- In terms of professional coverage
- In terms of continuity
- In terms of relational factors (between patient and employee)
- In terms of geography

How were patients’ needs addressed during the intervention?

How was the productivity of the unit affected during the intervention?

How could the intervention be improved to better fit the daily operations of your home care unit?

How do you perceive the culture for change in your workplace?

How did the intervention fit into the daily operations?

Did you experience any obstacles or challenges?

- In terms of tools
- In terms of the EPJ (helseplattformen)
- In terms of competing demands from management
- In terms of the overall workload

ADOPTION

How did you handle any obstacles or challenges?

Do you have any thoughts on how the distribution of roles regarding operational management affected the intervention?

What changes or adjustments did you need to make to carry out the intervention, and how were these needs followed up?

FIDELITY

How was it to follow the intervention as intended?
(Explanation of what it means to follow the intervention: using the tool to balance lists on a weekly basis.)

What considerations were made to comply with the intervention?

MOTIVATION TO CONTINUE/ MAINTENANCE

To what extent has the intervention become part of daily operations, and can you explain why?

- Can you say something about how this intervention could become part of daily operations?

Now that the intervention is over, can you reflect on your motivation to continue using it?

What would it take for you to continue using the intervention?

What would it take for such an intervention to be used in home care services in the future?
