# Supplementary material for: “It's a good idea, but…”: a qualitative evaluation of the GoldiCare intervention in Norwegian home care services
Source: Front Health Serv. 2025 Jan 20;4:1511772. doi: 10.3389/frhs.2024.1511772 (PMC11789199; doi:10.3389/frhs.2024.1511772)
Supplement: Supplementary file 3 [file Supplementaryfile3.pdf]

**Dimension in the analytical framework**	**Main analytical theme**
Acceptability	Good idea
	One more thing
Appropriateness	Appropriate time horizon
	Self-care ADL score does not sufficiently capture experience of physical demands
Feasibility	Cumbersome
	Adding mental load
	Challenges to the variation of work schedules
	Continuity of care
	Geographically based teams
	Availability of staff
Adoption	Leadership support
	Time to focus
	Teamwork
Fidelity	Varying incorporation
	'Goldicare pluss'
